# Adipose-Derived Stromal/Stem Cells from Large Animal Models: from Basic to Applied Science

**DOI:** 10.1007/s12015-020-10049-y

**Published:** 2020-10-06

**Authors:** Joanna Bukowska, Anna Zuzanna Szóstek-Mioduchowska, Marta Kopcewicz, Katarzyna Walendzik, Sylwia Machcińska, Barbara Gawrońska-Kozak

**Affiliations:** Institute of Animal Reproduction and Food Research, Polish Academy of Sciences, Tuwima 10, 10-748 Olsztyn, Poland

**Keywords:** Adipose-derived stem cells, Livestock animals, Animal models, Therapeutic application

## Abstract

Adipose-derived stem cells (ASCs) isolated from domestic animals fulfill the qualitative criteria of mesenchymal stem cells, including the capacity to differentiate along multiple lineage pathways and to self-renew, as well as immunomodulatory capacities. Recent findings on human diseases derived from studying large animal models, have provided evidence that administration of autologous or allogenic ASCs can improve the process of healing. In a narrow group of large animals used in bioresearch studies, pigs and horses have been shown to be the best suited models for study of the wound healing process, cardiovascular and musculoskeletal disorders. To this end, current literature demonstrates that ASC-based therapies bring considerable benefits to animal health in both spontaneously occurring and experimentally induced clinical cases. The purpose of this review is to provide an overview of the diversity, isolation, and characterization of ASCs from livestock. Particular attention has been paid to the functional characteristics of the cells that facilitate their therapeutic application in large animal models of human disease. In this regard, we describe outcomes of ASCs utilization in translational research with pig and horse models of disease. Furthermore, we evaluate the current status of ASC-based therapy in veterinary practice, particularly in the rapidly developing field of equine regenerative medicine. In conclusion, this review presents arguments that support the relevance of animal ASCs in the field of regenerative medicine and it provides insights into the future perspectives of ASC utilization in animal husbandry.

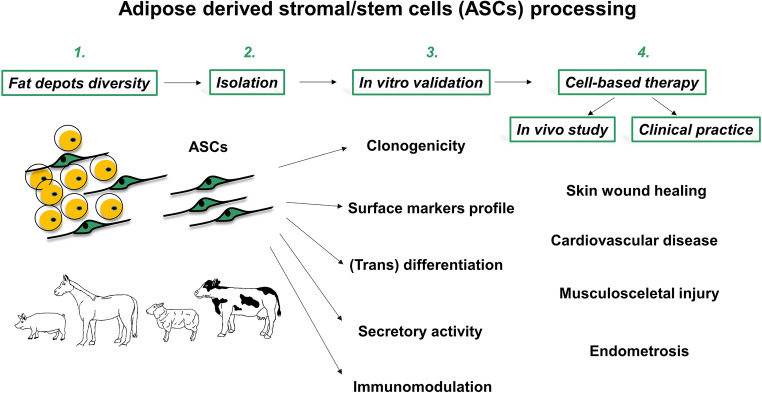

## Introduction

Mesenchymal stem cells (MSCs) can be isolated from a large variety of tissue sources, among which bone marrow mesenchymal stem cells (BMSCs), first identified in 1968 by Friedenstein et al. [1], have been the most extensively investigated. However, due to the limitations related to BMSC procurement, mostly reflected by the high risk of morbidity associated with the bone marrow aspiration procedure and the critically low yield of isolated MSCs (0.001%–0.01% of the harvested bone marrow cells), there has been a growing need to explore an alternative source of MSCs [2]. Discovery/appreciation of adipose tissue as an abundant source of MSCs led to overcoming the BMSC-associated obstacles and introduced a new type of MSCs named adipose-derived stem cells (ASCs) [3]. Indeed, fat tissue deposits are accessible, and they can be obtained from multiple sites in large quantities. In this context, one of the main advantages of ASCs over BMSCs is that adipose tissue can be easily harvested using minimally invasive procedures, such as liposuction and chirurgic interventions, that in humans are employed as methods for excess fat removal, and in the case of farm animals, adipose tissue can be obtained from slaughterhouses [4, 5]. Another biological feature that advocates for ASCs is their lifespan during in vitro culture. As demonstrated by Kern et al., BMSCs exhibited signs of cell senescence at passage 7, whereas ASCs can be cultivated up to passage 8 without any sign of decline [6]. Although significant differences have been reported between human ASCs and BMSCs, both populations demonstrate comparable characteristics in terms of morphology, surface markers, and differentiation potential [7–9]. Moreover, numerous similarities between these cells were confirmed based on transcriptomic analysis [10].

ASCs have received considerable attention over the past decades, mainly because of their therapeutic potential in different areas of applied science [11–13]. Although the initial method of cell isolation from adipose tissue was established using laboratory rodents [14–17], soon thereafter ASCs were isolated from human fat [3]. Human ASCs (hASCs) have now been extensively studied and are employed in preclinical and clinical studies [18, 19]. In recent years ASCs from domestic animals have gained increased attention, primarily because of the accumulating evidence demonstrating their remarkable plasticity, differentiation potential, expression of pluripotency markers, and immunomodulatory properties at levels comparable to those observed in hASCs [20, 21]. Together, these modalities strongly endorse farm animal ASCs as potential therapeutic agents that can be widely considered for clinical application in veterinary medicine. At present, many veterinary clinics take advantage of autologous or allogenic ASCs to treat various diseases in animals. In particular, cell-based therapies using ASCs are increasingly being reported in equine medicine because this field involves a large number of animals prone to frequent injuries to tendons, joints, cartilage, and bones (reviewed in [22]). Last but not least, an important reason to support the use of large animal models as a target for ASC administration is that they provide insights into disease mechanisms and models for determining the efficacy and safety of cell-based therapies. Among these, the animal model that shows results parallel to those expected in humans is pigs [23].

This review combines basic and applied science to discuss ASCs in the context of veterinary medicine. We describe the recent advances in studies of the source, isolation, and characterization of ASCs from livestock. Particular attention is directed toward the functional characteristics of cells that facilitate their application in large animal models for improving repair of multiple tissues in both humans and animals. In this review, we also provide an overview of the translational studies that demonstrate application of ASCs in some important pig models. Moreover, we evaluated the current status of ASC-based therapy in animals, particularly in rapidly developing equine regenerative medicine.

## Basic Biology of ASCs Isolated from Livestock

### Fat-Depot–Dependent Quantity of ASCs

The most abundant type of adipose tissue in adult mammals is white adipose tissue (WAT), which serves multiple functions, including energy storage and endocrine activity [24]. WAT is composed of a heterogeneous population of cells with the two main cell fractions being lipid-filled adipocytes and the stromal vascular fraction (SVF) that contains the population of ASCs [25]. Routinely, during cell isolation and culture procedures, ASCs are purified from SVF by adherence to plastic and further expansion in culture [25]. In mammals, WAT depots occupy different anatomical locations and demonstrate morphological and functional heterogeneity [26]. In the pig, abdominal and dorsal regions of subcutaneous fat have been the most frequently used source of ASCs [27–30]. There is only one report that refers to the caudal part of the body for ASC isolation [31]. Similarly, very few investigations have described pig ASCs (pASCs) collected from visceral or inguinal fat [32, 33]. In equine studies, similar to porcine studies, the large body of data has been obtained based on subcutaneous fat [20, 34–36]. However, the sites chosen for subcutaneous adipose tissue harvesting seem to be far more diverse than in other animal species and include the paracoccygeal region, supragluteal area, the region above the dorsal gluteal muscles, the tail base (tail head), and the area lateral to the insertion of the tail [20, 37–40]. However, other tissues, including intraperitoneal adipose tissue, omentum, hepatic falciform ligament and mesenteric lipomas, have also been reported as a source of ASCs [37, 41]. Interestingly, in other domestic animals, such as sheep and cows, data reveal the presence of ASCs in unique locations, such as the infrapatellar fat pad and the hoof interdigital region, respectively [42, 43].

In the current literature, there are limited comparative studies referring to cell yield or isolation method efficiency between different fat depots or different mammalian species [Table 1]. One example of comparative studies is that by Perruchot et al. [28]. Considering the developmental and functional link between adipose tissue and skeletal muscle [45], the authors compared pASCs isolated from subcutaneous dorsal adipose tissue (SCAT) and intermuscular (interM) fat, to cells collected from skeletal muscle. They showed a lower number of SVF cells isolated from interM fat depot compared to other examined tissues regardless of donor age (7-, 160-, 400-days old). Moreover, immunophenotypic analysis of SVF cells revealed that, whereas the proportion of CD90+ cells increased in interM fat and muscles in adult pigs, the pattern did not change with age in SCAT. However, another report by Niada et al. [44] demonstrated no differences in viability, proliferation, clonogenicity and adipogenic, osteogenic, or chondrogenic differentiation between pASCs from subcutaneous interscapular fat and buccal fat pads. Interestingly, this data supported future clinical applications of stem cells from buccal fat, the depot that is discarded tissue during cosmetic surgery for cheek reduction in humans [46]. Moreover, Burk et al. [38] found that adipose tissue provided a higher number of cells per tissue unit as compared to umbilical cord blood or flexor tendon. In addition, the culture time for primary cells (passage 0) was shorter for ASC (6 days) than for bone marrow MSCs (14 days).

**Table 1:** Yield of ASCs^a^ isolated from different fat depots in pigs and horses

Animal	Sex and Age	Fat depot	Number of cells	Unit(number of cells per)	References
Pig	Females and males,≥4–5 month-old	Subcutaneous interscapular fat (ScI-pASCs); Buccal fat pads (BFP-pASCs)	5.5 × 10^4^ (± 3.3 × 10^4^); 3.0 × 10^4^ (± 9.3 × 10^3^)	mL raw tissue	[44]
Pig	Females,7, 160, 400 days old	Subcutaneousdorsal adipose tissue (SCAT); Intermuscular (interM) adipose tissue	2.0 × 10^7^ (± 0.5 × 10^6^; 7 days old); 6.0 × 10^6^ (± 0.2 × 10^6^; 160 days old); 2.6 × 10^6^ (± 0.2 × 10^6^; 400 days old); 5.5 × 10^6^ (± 0.4 × 10^6^; 7 days old); 0.3 × 10^6^ (± 0.03 × 10^6^; 160 days old); 1.8 × 10^6^ (± 0.3 × 10^6^; 400 days old)	gram tissue	[28]
Pig	Not referenced	Caudal region	1.8 × 10^5^ (± 4.7 × 10^4^)	mL raw tissue	[31]
Horse	Median age 4.5 years	Supragluteal region	1.78 × 10^6^	gram tissue	[38]

^a^ASCs, adipose derived stromal/stem cells

### ASC Isolation and In Vitro Propagation

**Fig. 1:**
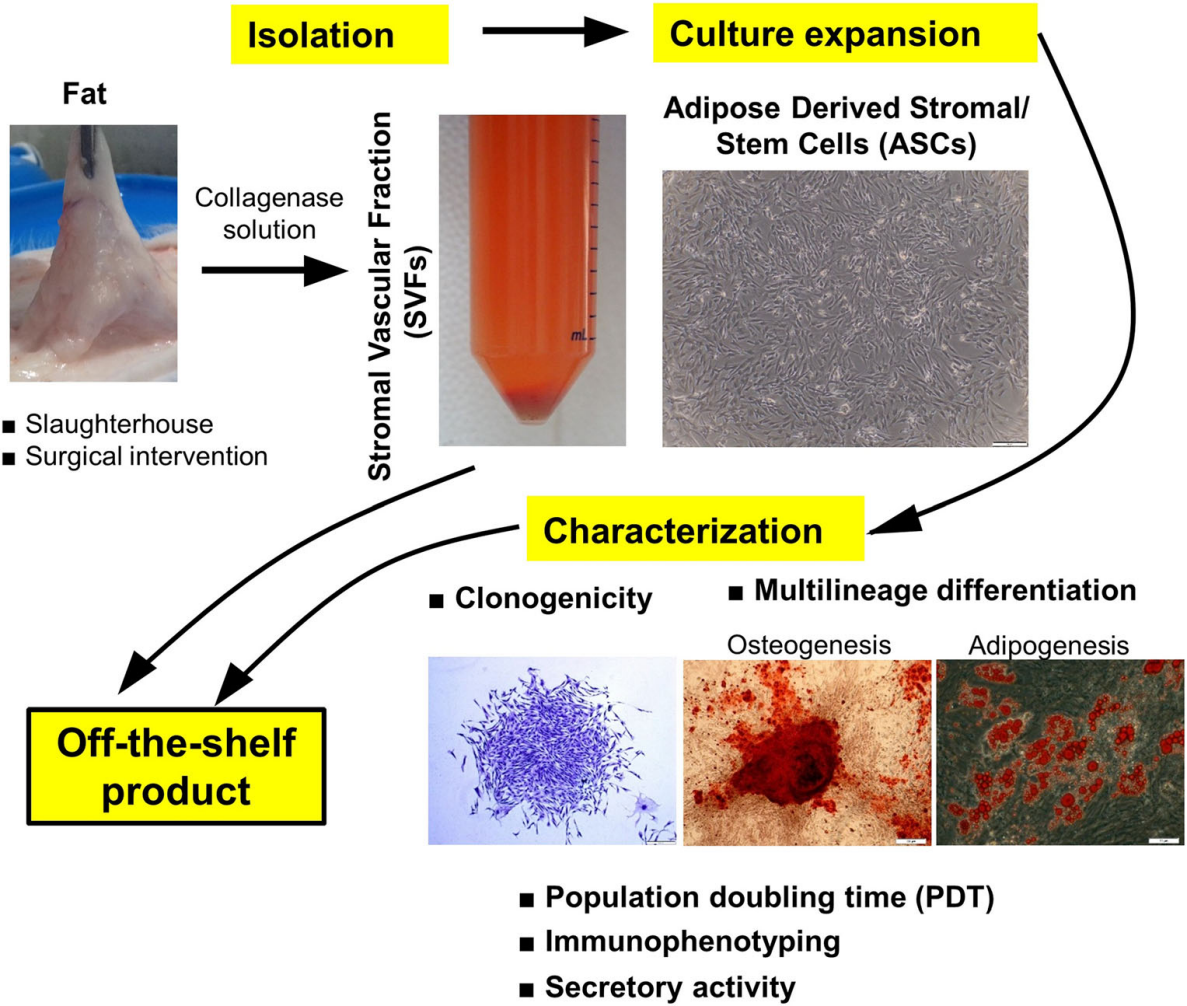
Schematic illustration depicting fat tissue processing, isolation of stromal vascular fraction cells (SVFs), and culture expansion of adipose-derived stromal/stem cells (ASCs). Verification of the functionality of SVFs/ASCs in vitro and the identification of their surface markers should be an obligatory step to approve cells as an off-the-shelf product

Rodbell and colleagues in the 1960s [14–17] pioneered the procedures for isolation of ASCs from adipose tissue by a series of experiments on rat epididymal fat. Despite a number of minor modifications made to the initial protocol through the years, the core method remains unchanged and relies on collagenase digestion at 37 °C with continuous agitation [47, 48]. Samples are then centrifuged in order to separate the floating population of mature adipocytes from the pelleted SVF. Finally, the resulting heterogenous SVF can be seeded into culture dishes in medium containing fetal bovine serum (FBS) to further expand the plastic-adherent population of ASCs [47] (Fig. 1). Consistently, numerous animal studies show that the collagenase concentration (primarily type I or II) varies between 0.01% and 0.2% with very strong representation of protocols demonstrating 0.1% as an appropriate dilution of the enzyme [21, 29, 49]. There are also reports describing a combined enzyme procedure composed of trypsin (*w*/*v*, 0.25%) and collagenase type I (w/v, 0.1% or 0.2%) that was used for ASC isolation from abdominal fat of cattle [50] and from ovine adipose tissue [51]. Medical products, including Matrase™, have also been reported to have been used [52].

ASC proliferation has been demonstrated to be species-specific and highly dependent on the culture medium used. Indeed, Schwartz et al. [29] found that population doubling time (PDT) of pig ASCs varied between 65 ± 7 h for cells cultured in medium supplemented with FBS and 54 ± 3 h in medium supplemented with serum substitute. Other studies showed that the PDT of pig ASCs was 54 ± 19 h in serum-containing medium [31]. A similar discrepancy has been observed in studies that used bovine ASCs [43, 53]. Independent studies from two laboratories have demonstrated that the PDT of cells from passage 1 to 5 extended and reached values of 16.44 ± 0.33 h and 22.06 ± 0.32 h, respectively, whereas other investigators have reported a constant PDT from passage 1 to 9 that equals 30 ± 1.9 h [43, 53]. Similarly, no differences in the proliferative ratio of ASCs were observed in ovine ASC cultures during progressive expansion up to passage 6 [42]. In horse ASCs, the PDT was shown to be 24.23 ± 4.45 h [54]. In contrast, others revealed that under comparable culture conditions the PDT of equine ASCs was 2.2 ± 0.2 days [40]. Although the proliferation of equine ASCs has been shown to be highly variable, there is considerable evidence that this function is greater in ASCs than in bone marrow MSCs [38, 40] or umbilical cord MSCs [38]. Moreover, studies by Marycz et al. [55] shed light on ASC functional characteristics in terms of health status of the animals. In this particular study, the authors prepared ASCs isolated from healthy donors and horses affected by equine metabolic syndrome (EMS), manifested by pathological obesity, hyperglycemia, hyperinsulinemia, and insulin resistance [39]. ASCs derived from EMS (ASC_EMS_) horses exhibited significantly decreased proliferation at 7 days of culture and required more time to double their population (73.75 ± 6.54 h), compared with ASCs from healthy donors, with a PDT of 51.12 ± 5.32 h [55]. In addition, ASCs_EMS_ exhibited a senescent phenotype manifested by β-galactosidase accumulation and displayed numerous degeneration-related indicators, such as increased amounts of nitric oxide (NO) and reactive oxygen species (ROS), reduced superoxide dismutase (SOD) activity, and a growing number of impaired mitochondria. Interestingly, in a further study, the same group demonstrated characteristics of equine ASCs as a function of age [34]. ASCs derived from middle-aged (5–15 years old) and aged (>15 years old) animals showed a typical senescence phenotype including, among other features, decrease in proliferation, elevated β-galactosidase activity, and an increased percentage of G1/G0-arrested cells. This association between animal age and ASC quality should be carefully considered during selection of the cell donors.

More recently, Arnhold et al. [37] provided complementary data on this topic and compared equine ASCs from three fat tissue sources: subcutaneous adipose tissue, retroperitoneal fat, and mesenteric lipomas, which are soft-tissue tumors. According to this study, ASCs derived from retroperitoneal fat showed increased viability and capacity to proliferate as compared to other ASCs, among which ASCs from lipomas exhibited the lowest levels of these functional characteristics [37].

In turn, Dariolli et al. [56] provided some important data regarding the effect of long-term cryopreservation in 10% DMSO on growth kinetics of pASCs. This study showed no difference in terms of PDL between thawed and freshly isolated pASCs, which were 64.26 ± 15.11 h and 62.74 ± 18.07 h, respectively. Likewise, based on β-galactosidase activity, senescence was comparable in both types of pASCs and did not exceed 10% at P10. Moreover, cryopreservation had no impact on the adipogenic and osteogenic differentiation of these cells. These findings provided evidence for feasibility and advantages of cryopreserved pASCs that can be considered for cell banking and future use in preclinical studies and therapeutic applications.

### Stemness-Related Markers

In 2013 the International Federation for Adipose Therapeutics and Science (IFATS) and the International Society for Cellular Therapy (ISCT), represented by Bourin and coworkers [25], provided guidance regarding the minimal criteria expected for ASCs. These properties have been broadly used as the gold standard in characterization of ASCs derived from both humans and animals.

Animal ASCs are commonly described based on their clonogenicity, immunophenotype, and trilineage differentiation potential into adipogenic, ostegenic, and chondrogenic lineages. Clonogenicity, described also as a colony-forming–unit fibroblast (CFU-F) [57], has been observed in a majority of ASC cultures regardless of donor species. Certainly, the potential to create colonies from a single cell depends on passage number. Having compared clonogenicity of pig ASCs at P0, P5, P10, P15, and P20, Tang et al. [58] showed a decline in the number of CFU-Fs in higher passages and demonstrated that the number of colonies at each passage was 1041 ± 19.3, 713 ± 22.1, 280 ± 16.7, 36 ± 2.9, and 0, respectively. In addition, in horse studies, the reduction in number of colonies was observed in ASCs from a lipoma and in cultures established from an EMS fat depot [37, 39]. It is noteworthy that da Silva Meirelles et al. [59] found a correlation between CFU-F number and blood vessel density. In this study, ASCs were isolated from horse subcutaneous fat of the tail head showed increased vascularity relative to the adipose tissue in the area lateral to the tail insertion. The CFU-F numbers per mg of adipose tissue were next examined as a function of the area occupied by blood vessels in each sample. The results revealed a strong correlation (*r*^*2*^ = 0.86) between these two modalities, supporting the earlier findings suggesting that the perivascular region can create the niche for adipose tissue stem cells [60]. However, it should be mentioned that the study was performed with a small number of replicates (*n* = 2) and without quantification of specific markers for endothelial cells or pericytes due to the lack of specific anti-horse antibodies and the lack of reactivity of anti-human antibodies [59].

ASCs are routinely characterized using a panel of surface markers or occasionally pluripotency-associated markers, including Oct4, Nanog, and Sox2, which have been shown to be expressed in pig ASCs [21, 30, 61, 62]. Despite any differences in species or fat depot, the immunophenotype of ASCs is uniformly consistent between laboratories [62–65]. Indeed, according to the current literature, markers that show strong positive expression on livestock ASCs are CD29, CD44, CD73, CD90, and CD105, whereas CD11b, CD14, CD34, CD45, and HLA-DR demonstrate low or lacking expression [62–65]. The expression profile of selected markers might change as a function of donor age. A study of pigs at different ages (7-, 160-, 400-days-old) revealed that the percentage of CD90 cells from the intermuscular region is elevated in adult animals (400-days-old) [28]. In horses, no significant differences were observed between ASCs and BMSCs. The percentage of CD29+ cells was 99% for ASCs and 90% for BMSCs; 87% of ASCs and 64% of BMSCs expressed CD105, whereas 99% of both demonstrated the presence of CD44 [66].

Being of mesodermal origin, the animal ASCs have demonstrated potential to differentiate into adipogenic, chondrogenic, and osteogenic lineages, regardless of species origin [20, 50, 52, 62]. Additionally, there is considerable evidence that, upon stimulation with specific induction media, ASCs are able to acquire the phenotype and function of cells belonging to ectodermal and endodermal lineages [30, 32, 65]. Principally, these abilities have accounted for application of ASCs in regenerative medicine. Comparative studies [20] revealed that adipogenic differentiation, evaluated based on accumulation of lipid droplets, was more noticeable in equine ASCs than in their human counterparts. A separate study demonstrated that the degree of osteogenic differentiation determined by extracellular calcium deposition was comparable in ASCs, BMSCs, and tendon MSCs, and subsequently, the process was more pronounced in these cells than in MSCs derived from umbilical cord blood or umbilical cord tissue from horses [38]. Furthermore, differentiation capacity changed with time in culture Zhao et al. [43] observed reduced adipogenesis and chondrogenesis of bovine ASCs at P5 relative to the cultures at P2. However, propagation during passages favored osteogenic differentiation as ASCs showed increased activity of alkaline phosphatase (ALP) at P5 relatively to P2 [43]. These findings are consistent with data obtained from pig ASCs demonstrating a decrease in adipogenic properties with increasing passage number (from P1 to P12), whereas the cells at late passage (P12) displayed enhanced osteogenesis compared with early (P1) and middle (P6) passages [33].

## Functional In Vitro Validation of Animal ASCs as an Introduction to Preclinical Application

Over the last few years, intensive research efforts have focused on developing cell-therapy products aiming to improve the cure of numerous disorders [67, 68]. Concomitantly, there has been debate in the stem cell field that questions the level of basic science knowledge necessary to enter clinical trials [69]. Certainly, the cellular strategies should reach the status of research-grade products prior to introducing a translational phase. There is a common opinion that the success of stem cells in regenerative medicine is profoundly dependent on their functional qualities, which should be thoroughly verified in vitro (Fig. 1).

There has been considerable progress in ASC propagation and differentiation protocols, anticipating the use of new treatments including growth factors, biomaterials, hypoxia, or even physical agents (e.g., magnetic fields) [36, 54, 65, 70–72]. These modalities provide improvements in ASC function and increase the chance of achieving beneficial outcomes in damaged tissue.

### Trans-Differentiation

In addition to the aforementioned properties of trilineage differentiation of adipogenic, chondrogenic, and osteogenic lines, animal ASCs were shown to possess the capacity to generate in vitro a vast array of cell types across germ line boundaries. Studies on pigs demonstrated that ASCs can be induced to produce hepatocytes in hepatocyte-differentiation medium [32]. Liu et al. [65] found that ASCs derived from piglet subcutaneous fat showed the ability to transdifferentiate into pancreatic islet-like clusters (PILCs), which was confirmed by expression of tissue-specific proteins, such as pancreatic and duodenal homeobox (Pdx-1), insulin and insulin gene enhancer protein (ISL-1). It is noteworthy that ELISA results showed that PILCs released high amounts of insulin into the culture medium upon glucose challenge. Moreover, a study using pig ASCs demonstrated myogenic and cardiomyogenic differentiation of pASCs, following incubation with culture medium supplemented with hydrocortisone or retinoic acid, respectively, together with other reagents [62]. In this case, long-term ASC culture (19 days) resulted in the formation of parallel, tightly packed structures resembling myofibers. Although the study showed lack of functional examination, immunofluorescence staining using human antibodies detected the presence of muscle and cardiac proteins, including α-sarcomeric actin, myogenin, myod1, desmin, cardiotin, and GATA-6, in ASC cultures [62]. Given the fact that biomedical research has employed ASCs in the area of myocardial dysfunction [73], such investigations can shed light on possible utilization of ASCs in veterinary practice. Furthermore, Song et al. [30] demonstrated the potential of pASCs for in vitro oogenesis. Indeed, after introduction of differentiation medium, ASCs exhibited formation of oocyte-like precursor cells by day 12. These observations were evaluated based on morphological changes displaying the formation of thecal, granulosa, or cumulus-like cells, and they were confirmed at the level of marker mRNA expression, including *Oct-4*, *growth differentiation factor 9b* (*GDF9b)*, *c-Mos*, *Vasa, deleted in azoospermia-like (DAZL)*, *zona pellucida C (ZPC)*, and *follicle-stimulating hormone receptor (FSHR)* [30].

Significant advances in the field of animal ASCs were provided by establishing pig induced pluripotent stem cells (piPSCs) from ASCs [58, 74]. Tang et al. [58] used retroviral transduction to reprogram pASCs and BMSCs. The reprogramming efficiency was comparable in both type of cells. Although the cells were not completely reprogrammed, they showed embryoid body formation and expressed *glial fibrillary acidic protein (GFAP)*, *alpha fetoprotein (AFP)*, and *bone morphogenetic protein 4* (*BMP4)* mRNA, which are relevant to the ectoderm, endoderm, and mesoderm, respectively. In a separate study, cell reprogramming was accomplished using doxycycline (DOX)-inducible lentiviral vectors encoding Yamanaka factors (Oct4, Sox2, c-Myc, and Klf4) and a unique culture system with no feeder layer and serum supplementation [74]. At a functional level, successful reprogramming of naive-like piPSCs was confirmed by differentiating into cell types of all three germ layers in vitro and in vivo. Because iPSCs can further serve as an alternative to embryonic stem cells (ESCs) facilitating formation of any germ layer, they might be considered “personalized” cell products.

### ASC Secretory Activity

A mechanism by which MSCs improve tissue repair includes both differentiation and paracrine signaling [75, 76]. However, because cumulative data on laboratory rodents indicated that the levels of improvement achieved with MSC application do not always correspond with the levels of cellular engraftment, it was assumed that the paracrine activity of MSCs is the primary mechanism accounting for their beneficial effects in responses to injury [75, 76]. This concept was further supported by studies demonstrating that not only MSCs alone but also MSC-conditioned medium has the ability to enhance tissue repair [77, 78].

While the trophic effects produced by the secretion of bioactive factors by MSCs have been widely reported in human and mouse (reviewed in [79, 80]), there is a limited number of studies showing the analogous properties of ASCs derived from farm animals. Pascucci et al. [81] demonstrated that equine ASC monolayers constitutively produce membrane vesicles (MVs) that mediate angiogenesis in vitro. The observation of MVs’ pro-angiogenic function came from the migration (scratch) assay of equine vascular endothelial cells (E-VECs) and rat aortic ring assays, both performed in culture medium supplemented with MVs. It was revealed that exposure to MVs significantly promotes E-VEC migration relative to cells treated with control medium. Likewise, supplementation with MVs increased neovessel formation from aortic rings as compared with control, which showed minimal or lack of vessel formation. Sprouting of microvessels that was maximal after 7 days of culture and exhibited complete structural organization have led the authors to suggest that the MVs probably carry more than one angiogenic molecule [81]. Another study by Klymiuk et al. [82] provided further evidence of exosome secretion by equine ASCs. Although the authors did not examine the exosomes with regard to their function, their technical report described vesicle isolation from ASC supernatants and their characterization, providing an introduction to later therapeutic usage [82].

### Immunomodulatory Properties of ASCs

One of the most clinically relevant properties of ASCs is their immunomodulatory capacity, as has been shown over the years in numerous experimental models [83–85]. In general, MSCs are widely described as being strongly immunosuppressive (reviewed in [86]). The ability to direct inhibition of the immune response by their paracrine factors might, in part, explain the reparative function of human ASCs observed in a variety of injury models [83]. To date, only a few studies have been conducted to investigate the immunomodulatory competence of farm animal ASCs [87, 88]. Holt et al. [88] showed that equine ASCs cultured in direct cell-cell contact with T cell-enriched peripheral blood mononuclear cells (PBMCs) caused inhibition of T cell proliferation at baseline and upon stimulation by alloantigen or mitogen phytohemagglutin (PHA). Moreover, treated ASCs secreted significantly more IL-6 and prostaglandin E2 (PGE_2_) than stimulated T cells. In contrast, ASCs decreased the levels of pro-inflammatory cytokines, such as tumor necrosis factor alpha (TNFα) and interferon gamma (IFNγ) produced by activated T cells. It is noteworthy that this work compared the immunomodulatory properties of ASCs and MSCs derived from other tissues including bone marrow, umbilical cord blood, and umbilical cord tissue. In addition to the differences in NO production, the authors revealed qualitative similarities among all investigated types of MSCs. In a further study, the same authors found that the immunomodulatory properties of equine MSCs might be determined by tissue source. For instance, ASCs and umbilical cord tissue inhibited T cell proliferation through the induction of lymphocyte apoptosis while bone marrow and cord blood caused lymphocyte cell cycle arrest [88].

In order to study the immunomodulatory role of ASCs, Falomo et al. [89] demonstrated an interesting approach with the use of allogenic ASCs seeded on mare endometrial biopsy explants. Quantitative PCR analysis of explants incubated for 3 days with ASCs showed reduction of *IL-1β, IL-10, TNFα*, and *IL-1RN* mRNA, whereas expression of *IL-6* and *IL-8* mRNA increased. Considering the fact that IL-1β and TNFα are recognized as pro-inflammatory cytokines, whereas IL-10 and IL-1RN show anti-inflammatory properties, these results suggest that ASCs might exert both positive and negative effects on endometrium in vitro [89].

With this background showing pro- and anti-inflammatory properties of equine ASCs, it can be presumed that immunosuppression is not an intrinsic, constitutive property of stem cells, but rather, the immunomodulatory abilities of ASCs are created by numerous independent stimuli. Studies performed by Bunnell et al. (reviewed in [86]) on human BMSCs led to the establishment of a new paradigm in MSC biology, which premised that MSCs, like monocytes, undergo polarization mediated by downstream Toll-like receptors (TLRs) into either a pro-inflammatory (MSC1) or an anti-suppressive reparative (MSC2) phenotype. This concept might explain the ability of ASCs to acquire disparate immune-modulating activities.

### ASC Culture Medium Supplementation

Numerous studies in horses have examined the effect of medium supplementation in order to improve functional characteristics of ASCs. Del Blue et al. [70] showed that autologous platelet lysate (PL) significantly promoted proliferation of ASCs during 96 h of culture. To clarify, two different types of PL were tested in this study: rich PL that contained 41.0 mg/mL protein and poor PL in which the protein content reached 20.5 mg/mL. Although medium supplementation with both PLs stimulated ASC proliferation, the rich PL exerted a greater effect on cell growth. Interestingly, according to the authors, protein concentration in both PLs was lower than that indicated in typical batches of fetal serum. In this line, the PL appeared to be an appropriate substitute for commercially available serum [70]. This study was further extended by Castro et al. [54] who found that application of vitamin C (0.1 mM) and PRP (5% *v*/v; PRP) alone, but primarily in combination, promoted ASC differentiation toward osteogenic and chondrogenic lineages, as demonstrated by histological examination of calcium deposits and glycosaminoglycans (GAGs) and confirmed based on marker gene analysis [54]. In sheep, it has been observed that among different factors, including basic fibroblast growth factor (bFGF), bone morphogenetic protein 2 (BMP2), and NEL-like molecule 1 (NELL1), added to the osteogenic induction medium at four different doses (1, 10, 50, and 100 ng/mL) each, only bFGF at the concentration 10 ng/mL enhanced osteogenic differentiation of ASCs [90]. Interestingly, Kalaszczynska et al. [91] demonstrated differences in osteogenic differentiation capacity between human and ovine BMSCs, and ASCs. Although mineralization of human cells was achieved in standard osteogenic medium supplemented with β-glycerophosphate, the commonly used organic source of phosphate ions, their ovine counterparts were unable to enter osteogenic induction upon the same treatment. Noticeable mineralization of the extracellular matrix of ovine BMSCs and ASCs was observed upon the addition of an inorganic source of phosphorus ions (NaH_2_PO_4_) to the culture media.

### Three-Dimensional (3D) Scaffolds

There is significant interest in taking advantage of biomaterials and 3D scaffolds in order to improve the desired functions of ASCs, such as prolonged survival, more rapid proliferation, or enhanced differentiation. Liu et al. [65] investigated the use of chitosan-coated plates for differentiation of pASCs into PILCs and found that chitosan accelerated cellular differentiation, and the cultures developed PILCs as early as day 3, whereas on uncoated plates this process was delayed until day 12. Additionally, immunofluorescence staining revealed that chitosan upregulated insulin production on day 3, compared to day 6 on regular plates. At the molecular level, it has been demonstrated that the scaffold substantially increases the expression of β-cell differentiation-associated transcripts, including *PAX-4*, *Pdx-1*, *glucokinase*, and *insulin*. In another study, Kisiday et al. [72] showed that equine ASCs encapsulated in agarose or self-assembling peptide hydrogels responded to TGF-β1 stimulation as reflected by accumulation of extracellular matrix (ECM) components such as proline, sulfate, and GAGs. It should also be noted that the efficiency of ECM deposition was higher for ASCs loaded into peptide hydrogels than for those seeded on agarose. Moreover, this effect was amplified in the case of BMSCs relative to ASCs [72]. There are also data demonstrating that collagen scaffolds support trilineage differentiation of horse ASCs and BMSCs [66]. More interestingly, the cells were entrapped within the scaffold and cultured in a perfusion bioreactor system, which provided a controlled environment for cell loading and resulted in higher seeding efficiency than with standard static cell loading methods. Therefore, this technique may be considered a tool for large-scale production of cell-scaffold constructs/implants for the purpose of regenerative medicine [66].

### Other Factors

It should be emphasized that there are some alternative treatments to modify the behavior of ASCs. For example, Raabe et al. [36] demonstrated that extracorporeal shock-wave therapy (ESWT), which is used in equine veterinary practice for improving the healing of bone, tendon, or cartilage, exerts an effect on the cellular level and positively alters equine ASCs. Indeed, experimental conditions that relied on 9 pulses of 1000 shock waves and 3 pulses of 2000 shock waves led to significant increase in ASCs proliferation and contributed to more pronounced trilineage differentiation compared with the control untreated group. Moreover, ESWT treatment promoted expression of gap-junction protein *connexin 43* (*Cx43*) mRNA. The mechanisms underlying the observed changes were related to activation of extracellular-signal–regulated protein kinases 1 and 2 (Erk1/2) [36]. Similarly, Maredziak et al. [35] exposed equine and canine ASCs to a static magnetic field (MF) with intensity of ~0.5 T. The study showed that equine, unlike canine, ASCs had a shorter doubling time than untreated control cells. Additionally, cells derived from equine adipose tissue demonstrated numerous MVs on their surface in response to MF. As shown in human ASCs, MVs carry a variety of bioactive molecules that enhance the recovery of damaged tissue [92, 93]. Hence, these studies suggest that treatment with MF stimulates the secretory function of ASCs, supporting the paradigm that ASCs exert their effect on the surrounding environment via paracrine action. Due to the fact that damaged tissues are often characterized by limited oxygen or nutrient deprivation (reviewed in [94]), much attention has been paid to identifying strategies that lead to improving ASC survival in this environment. Along this line, preconditioning with hypoxia was shown to play a protective role in human stem cells, support their functions, and improve their therapeutic effect in vivo] [95–97]. Shell et al. [98] demonstrated that equine ASCs cultured in the presence of 3% O_2_ showed reduced proliferation compared to normoxic control conditions (21% O_2_)_._ Furthermore, whereas hypoxia improved adipogenesis and chondrogenesis, the condition reduced osteogenic differentiation capacity [98].

## In Vivo Study with Domestic Animal ASCs

Although laboratory rodents still remain the most significant and widely used experimental animals, large animals have become increasingly important in developing biomedical research. Considering the body size, specific anatomical and physiological characteristics, or the tendency to suffer from similar diseases as human (e.g., type I diabetes, hypertension, allergies, cancer, epilepsy, myopathies; reviewed in [99]), large animal species are more suitable to mimicking human clinical conditions. Moreover, from the perspectives of regenerative medicine and cell-based therapies, another important advantage of large animals is their relatively long life span, which allows monitoring of the long-term effects of a given therapy and answers questions regarding the benefits and potential obstacles of a treatment.

**Fig. 2:**
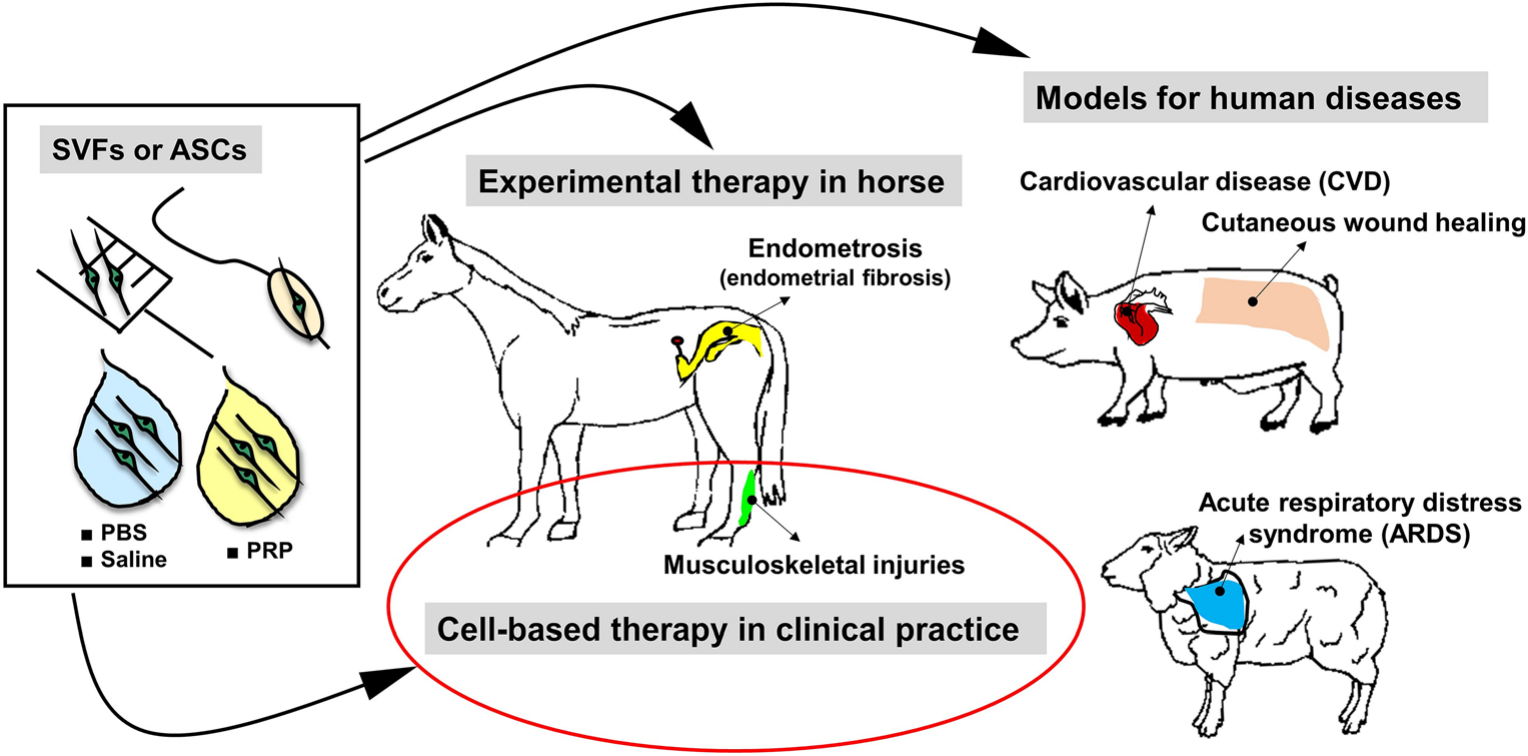
Utilization of ASCs and/or SVFs in translational research with pig, horse, and sheep models of human disease, and therapeutic applications of these cells in veterinary practice. Routinely, ASCs/SVFs are suspended in phosphate-buffered saline (PBS), 0.9% sodium chloride (saline) or platelet-rich plasma (PRP). Different routes of administration of these cells are practiced, including intra-tissue or intravenous injections (musculoskeletal injury treatment, cutaneous wound healing, acute respiratory distress syndrome), intracoronary delivery using balloon angioplasty catheter (cardiovascular disease), or insemination catheter (endometrosis). Although ASCs/SVFs have been applied in pigs and sheep to treat induced injuries, to serve as models of human trauma, ASCs-based therapy in horses has been used in spontaneously occurring clinical cases of musculoskeletal injuries

The selection of an appropriate animal model for translational research/cell-based therapy depends on a number of factors, including availability, ease of handling, anatomical and functional resemblance to humans, and specificity of the problem that the study aims to address. In view of current literature demonstrating the effect of MSCs on a broad range of diseases, there is considerable evidence indicating some preferences in the choice of the most suitable species for a study of interest (Fig. 2). For example, pigs have been extensively used to study the wound healing process due to the numerous similarities of pig skin to human skin, such as relatively thick epidermis, dense elastic fibers in the dermis, similar biochemical structure of collagen, sparse hair, and epidermal turnover of approximately 30 days [113, 114]. Importantly, pig wound models have been regarded as the most appropriate to model cutaneous wound healing in humans (reviewed in [115]). In a review, Sullivan et al. [116] provided comparison of the data that documents wound therapies in different animal models and found that pig models were 78% consistent with human studies; this record exceeded that of other species, which overlapped with human data by only 53%. In another review, Seaton et al. [115] presented an in-depth report of a generation of multiple pig wound-healing models and their use to study both normal and pathological wound healing, including chronic non-healing wounds, diabetic wounds, burns, wound infections, and hypertrophic scars. This confirms that pig models have been increasingly appreciated in the investigation of healing of wounds of numerous etiologies and that all have served as clinically relevant models. The second large research area in which pigs have often been used as a model is cardiovascular disease, as this species parallels favorably to human cardiovascular anatomy and physiology with respect to distribution of coronary arteries, ventricular performance, cardiac metabolism, electrophysiology, and collateralization after acute myocardial infarction [117]. In contrast, the horse is already established as an animal model for focal cartilage injuries and osteoarthritis (reviewed in [118, 119]). An important advantages of horse joint models are their sheer size, which allows for easy manipulation and exploration, and cartilage thickness and composition, which share close similarities with human articular cartilage [120]. The horse has also been shown to be an advantageous model of tendon and ligament injuries, due to the fact that many spontaneous injuries observed in horses are similar to those reported in human athletes (reviewed in [121]). Although a number of preclinical studies have investigated the use of ASCs in animal models of diseases, data on the application of ASCs in farm animals other than pigs and horses (e.g. sheep and cattle) are limited. Of note, Ihara et al. [52] used an ovine model of acute respiratory distress syndrome (ARDS) induced by smoke inhalation to estimate the efficacy of intravenously administrated ASCs. It has been shown that ASCs delivered at a dose of 2.0 × 10^8^ cells, remarkably reduced pulmonary microvascular hyperpermeability, significantly ameliorating pulmonary gas exchange and improving the oxygenation index. Additionally, considering the fact that the treatment was well tolerated by animals, as negative hemodynamic changes did not occur, the study suggested that ASC-based therapy might provide a safe and efficient alternative to treat patients suffering from ARDS [52] [Table 2]. Although the use of cattle as a model system has provided a remarkable contribution toward the advancement of biomedical research (reviewed in [122, 123]) there is a lack of published pre- and clinical reports specifically validating ASCs as a therapeutic means in this species. Nevertheless, existing studies demonstrating protocols of efficient ASC isolation from bovine fat, the in vitro approval of cell functionality, and the knowledge obtained from non-bovine large models, creates a path toward developing stem cell therapies in cattle [43, 50, 53]. Last, but not least, is the fact that while veterinary patients are increasingly recognized as critical translational models of human diseases, some cell-based therapies are dedicated exclusively to providing cures for animal disease, with no secondary intention. MSC-related therapies have been documented as being used as instruments to address orthopedic conditions in horses [70]. Currently, a large proportion of pet owners and their veterinarians worldwide have turned to such treatment options, making stem cell-based therapies a reality in veterinary medicine.

**Table 2:** Summary of in vivo application of ASCs^a^ in experimental disease models and spontaneous clinical cases in pigs, mini pigs, horses and sheep

Animal	Specific model	ASC treatment	Follow up	Outcome	References
Pig	Wound healing in diabetic pigs (full-thickness circular wounds)	Allogeneic ASCs: 5 × 10^6^, 1 × 10^7^; Allogeneic EC^b^/ASCs: 5 × 10^6^, 1 × 10^7^; ASC-CM^c^ : topical application in 2 mL serum-free medium	Up to 28 days	Cellular therapy and topical application of ASC-CM caused acceleration of wound closure rates, increased angiogenesis, and attenuated acute inflammation	[100]
Pig	Wound healing (full-thickness circular wounds)	Allogeneic ASCs: 3 × 10^6^/cm^2^, 1 × 10^6^/cm^2^, 3 × 10^5^/cm^2^	1 and 2 weeks	High-dose ASCs accelerate wound contraction, enhance neovascularization, and increase α-smooth muscle actin expression;medium-dose improved Col1:Col3 (collagen 1:collagen 3) ratio	[101]
Pig	Wound healing (deep partial-thickness burn wounds)	Allogeneic ASCs: 2 × 10^6^; Allogenic fat	Up to 10 weeks	Reduction in scar size;decreased expression of *BARX2 *transcript, which encodes protein involved in myofibroblast migration and differentiation;upregulation of *SPRY-2* mRNA encoding protein involved in FGF2^d^ receptor ligand binding and activation; diminished vessel presence	[102]
Mini pig	Wound healing (partial-thickness wounds produced by dermatome)	Allogeneic ASCs (or BMSCs): 1 × 10^6^; Wounds were dressed with bismuth gauze and fibrin sealant	Up to 21 days	Improvement in scar cosmetic appearance (vascularity, pigmentation, pliability, and height) and faster re-epithelialization compared to saline controls	[23]
Pig	Delayed wound healing (full-thickness wounds made in irradiated skin)	Allogenic ASCs: 4 × 10^6^ (1.8 × 10^6^ cells/cm^2^) in saline solution or PRP^e^	Up to 21 days	Faster re-epithelialization and increased microvessel densities. However, the healing enhancement depends on the combination of ASCs and PRP, as neither ASCs nor PRP alone showed an effect	[103]
Pig	Wound healing (full-thickness wounds)	Allogenic ASCs: Topical application of ASCs in PRP or PPP^f^	Up to 21 days	Improvement in scar cosmetic appearance and increase in microvascular density in a group receiving ASCs+PRP; no effect of ASCs on wound closure rate	[104]
Pig	Acute myocardial infarction (AMI) induced by occlusion of the mid LAD^g^ with angioplasty balloon for 3 h	Autologous SVFs: 1.5 × 10^6^ cells/kg injected into the infarct artery	8 weeks	Functional improvement manifested by reduction of myocardial perfusion defect and increase in myocardial salvage index and ejection fraction.Structural improvement reflected by increased wall thickness of the infarct and border zone and increased capillary density in the border zone	[105]
Pig	AMI induced by occlusion of the mid LAD with angioplasty balloon for 180 min	Autologous ASCs (or BMSCs): 2 × 10^6^ in 4 mL PBS	4 weeks	Reduction of absolute and relative perfusion defect,increased left ventricular ejection fraction,increased relative thickness of the ventricular wall in the infarction area, andimproved vascular density of the border zone	[106]
Pig	AMI induced by placing an angioplasty catheter into the mid LAD for 90 min	Autologous ASCs: 1.6 ± 1.1 × 10^7^ intracoronary delivery; 2.3 ± 0.8 × 10^7^ transendocardial delivery	3 weeks	Increased vascular density following intracoronary ASC administration	[107]
Mini pig	AMI induced by placing an angioplasty catheter into the mid LAD for 120 min	ASCs expressing green fluorescent protein (GFP): 2.13 ± 0.42 × 10^8^ transendocardial delivery	3 months	Increased angiogenesis and vasculoneogenesis andimprovement in heart remodeling (increased expression of TIMP^h^1, 2 but decreased MMP^i^2 activity)	[108]
Horse	Tendonitis (spontaneously occurring)	Allogenic ASCs: Delivered in association with PRP or PPP	Over 23 weeks	Of the 16 treated horses, 14 recovered and regained activity	[70]
Horse	Superficial digital flexor tendonitis (SDFT) (spontaneously occurring)	Allogenic ASCs: 2.0 × 10^6^ in 2–6 mL of PRP	24 months	Improved echogenicity of the tendon structuresstarting from 60 to 90 days after treatment shown by ultrasound images;functional recovery and return to previous level of activity observed in 89.5% of the horses, while re-injury rate occurred in 10.5% animal patients	[109]
Horse	SDFT (spontaneously occurring)	ASCs: 1.0 × 10^6^ in 5–10 mL of PRP	Up to 9 months	Increase in echogenicity, intralesional, and an array of grade 2 according to the tendon fibers by Reef at 30 days after transplantation;the alignment of tendon fibers reached grade 1 according to Reef with decrease in the size of the lesion after 60 days;full alignment of tendon fibers at 120 days,seven of nine horses resumed their normalathletic activity	[110]
Horse	Endometrosis (spontaneously occurring)	ASCs fluorescently labeled: 2.0 × 10^7^ in 20 mL of sodium chloride 0.9%	Up to 21 days	Detection of labeled ASCs in both uterine horns and uterine body;cells homed into periglandular space and uterineglands	[111]
Horse	Endometrosis (spontaneously occurring)	Allogenic ASCs fluorescently labeled: 2.0 × 10^7^ in 20 mL of sodium chloride 0.9%	Up to 60 days	Diminished expression of vimentin, α-SMA^j^ and cytokeratin 18 that correlated with positive histological changes in endometrial biopsy samples	[112]
Sheep	Acute respiratory distress syndrome (ARDS) (induced by smoke inhalation)	ASCs: 2.0 × 10^8^ in 200 mL of PlasmaLyteA	48 hours	Reduction of pulmonary microvascular hyperpermeability;increased oxygenation index and pulmonary gas exchange	[52]

^a^ *ASCs* adipose derived stromal/stem cells, ^b^ *EC* endothelial cells, ^c^ *ASC-CM* adipose stem cell conditioned medium, ^d^ *FGF2* Fibroblast growth factor 2, ^e^ *PRP* platelet-rich plasma, ^f^ *PPP* platelet-poor plasma, ^g^ *LAD* mid-left anterior descending artery, ^h^ *TIMP* tissue inhibitor of metalloproteinases, ^i^ *MMP* matrix metalloproteinase, ^j^ *α-SMA* smooth muscle actin alpha

Nevertheless, it should be noted that many authors focus their attention on the fact that in many cases stem cell therapies are applied in veterinary patients without relevant preclinical studies and are not controlled by regulatory agencies. This liberal approach has led to raising the general expectancy for regulations and guidelines to ensure standardization and quality assurance of cellular therapies being applied in veterinary practice (reviewed in [124, 125]).

### Wound Healing in Skin

With regard to large animal models, particularly pigs, ASCs and their potential to improve cutaneous wound healing have been investigated in several studies [23, 100–104] [Table 2]. James et al.] [101] performed a dose-response study of allogenic porcine ASCs delivered into four-centimeter circular full-thickness excisional wounds. They found that a high dose of ASCs (3.0 × 10^6^ cells/cm^2^) at 2 weeks post-injection accelerated wound contraction, increased neodermal thickness, and enhanced neovascularization when compared with lower-dose ASCs and saline-injected control wounds. It should be emphasized that because PKH26-labeled fluorescent ASCs were used, it was possible to identify their transplantation pattern in the healed skin. ASCs, however, were integrated into the deep neodermis at 1 week, but the signal weakened at 2 weeks post-delivery [101]. Perhaps this indicated paracrine signaling from injected ASCs, as it has been extensively reported in terms of wound healing in rodent models [92, 126].

As mentioned earlier, there are substantial arguments favoring some special pretreatment with ASCs, or the use of ASCs combined with numerous factors that might augment their engraftment and functions, and lead to potential ASC amplification to improve wound healing. In the work performed by Blanton et al. [104], ASCs were mixed with PRP or platelet-poor plasma (PPP), and then applied to full-thickness excisional wounds made on the dorsal skin of female Yorkshire pigs. Gross appearance at 3 weeks after wounding demonstrated a trend toward improved cosmetics in all experimental groups; however, only ASCs applied together with PRP exhibited significant improvement in the qualitative scoring scale. Likewise, vessel densities increased in samples receiving ASCs in association with PRP. This in vitro approach demonstrated an increased release of vascular endothelial growth factor (VEGF) from combined ASC and PRP cultures, indicating that ASCs enhance the process of healing, in part via contribution to revascularization, when provided with a fibrin matrix that is rich in complementary trophic factors [104]. In a separate study, Hadad et al. [103] employed a similar ASC delivery strategy to treat wounds in irradiated skin in a delay model of full-thickness injury of Yorkshire pigs. In this particular injury model, pigs received a single 20-Gy radiation dose to an 18 × 40–cm field of the dorsal skin. Seven weeks after the treatment, wounds were created in the irradiated area and subsequently ASCs suspended in PRP (or both treatments separately) were delivered to the wounds. Results revealed that the combination of ASCs and PRP was superior to all treatments in accelerating the rate of wound contraction and re-epithelialization. Furthermore, based on an immunohistological examination of α-smooth muscle actin (α-SMA) expression in biopsy specimens collected at 21 days post-injury, a significant increase was observed in the microvessel density in healed wounds that had been treated with the combination of ASCs and PRP, compared to those treated with saline solution [103].

Application of ASCs was also examined in a pig model displaying some wound healing pathologies including hypertrophic scarring [102] and diabetic wounds [100]. Rapp et al. [102] adapted a burn model of domestic pigs to determine whether autologous ASCs or intradermal delivery of autologous fat improved the process of healing by reduction of hypertrophic scar formation. In this model, mature wounds at 10 weeks post-injury were treated with saline, autologous ASCs, or fresh lipoaspirate. Scars injected with autologous fat or ASCs showed significant reduction in volume as compared with the saline-treated and unburned control group. However, neither ASCs nor lipoaspirate showed significant improvement in biochemical properties of scars 10 weeks post-treatment. In addition, while erythema was increased in all treated animals, the increase was statistically significant for scars that had been treated with ASCs or fat. On the other hand, RNA sequencing data demonstrated that, among 148 identified genes, as many as 99 showed significant differences in their expression across the treated group, compared with untreated scars. Of particular interest, genes with significantly altered expression upon ASC or lipoaspirate delivery, such as *BARX2* and *SPRY-2*, are known to contribute myofibroblast function. These molecular results provided strong indications that the treatment of hypertrophic scars with ASCs or fresh adipose tissue may lead to changes in regulatory pathways involved in wound healing dynamics that attenuated formation of hypertrophic scars [102].

Given the fact that impaired healing of diabetic wounds is a serious medical problem worldwide, and that so far none of the current research using mouse models has provided preventative or therapeutic cues, there is significant interest in finding ways to treat this type of injury. A porcine model that addresses this clinical problem was presented by Irons et al. [100] and relies on streptozotocin-induced diabetes followed by full-thickness excisional wounding of the back skin in female Yorkshire pigs. In this study, wounds were either injected with ASCs or endothelial-cell-differentiated ASCs (EC/ASCs) at a dose of 5 or 10 × 10^6^ cells, or they received topical application of conditioned medium from ASC (ASC/CM) or human umbilical vein endothelial cells HUVEC (HUVEC/CM) cultures. Of note, wounds subjected to cellular treatment received a repeat injection of one half of the initial dose on day 15, whereas those assigned to the topical CM therapy were covered with fresh CM every 3 days. The results showed an increase in wound closure rates upon treatment with both cells and CM at various time points during the post-wounding period of 28 days. Histological examination of tissue sections revealed a decrease in the acute inflammation scores in wounds receiving cell-based and CM therapy; however, the chronic inflammation scores were similar between the treated and control groups. Furthermore, no statistically significant differences were observed in the expression of angiogenic (CD31, platelet-derived growth factor [PDGF]), endothelial nitric oxide synthase [eNOS]) and pro-inflammatory (TNFα, IL-1β) genes at 28 days post-treatment. However, at the protein level, a significant decrease in TNFα was found in the wounds treated with ASC/CM and EC/CM. Overall, despite the fact that the general findings of these studies remain partially inconclusive and reinforce the need for further research using this particular wound model, there is strong evidence that employing CM provides a promising method to bypass the need to deliver ASCs to improve the healing outcome [100].

### Cardiovascular Disease

Cardiovascular disease (CVD) is the primary cause of morbidity and mortality worldwide with a recognized set of risk factors including obesity and sedentary lifestyle [127, 128]. The significant number of deaths associated with CVD result from heart failure due to ischemic events such as myocardial infarction (MI). Because the heart demonstrates limited reparative capacity, the restoration of cardiac function after an MI is challenging [129]. This is a major reason why the current revascularization procedures have provided modest regeneration effects, and it reinforces the need for exploration of alternative therapies for treatment of damaged myocardium (reviewed in [130]). Recently, numerous preclinical studies involving pigs have been conducted to analyze the efficacy, safety, and behavior of ASCs in the treatment of acute myocardial infarction (AMI) [105–108] [Table 2]. Valina et al. [106] induced an experimental AMI model by 3-h balloon angioplasty of the mid-left anterior descending artery (LAD), and then using the same balloon catheter, they performed an intracoronary injection of 2 × 10^6^ autologous cells (ASCs or BMSCs at a dose of 6 × 10^4^ cells/kg) in 4 mL phosphate-buffered saline (PBS). Four weeks after the onset of the experiment, nuclear cardiac imaging revealed significant functional and structural coronary improvements resulting from ASC and BMSC treatment. Specifically, both cell types increased absolute myocardial salvage as compared to the control PBS-treated group. Significant improvements in the motion of the left ventricular wall and its relative thickness in the infarction area were observed in the ASC-treated group. The presence of ASCs also led to increased vascular density in the border zone between infarcted and non-infarcted myocardium that was assessed based on immunofluorescence staining of von Willebrand factor (vWF). Delivery of BMSCs had a similar effect on capillary density; however, the data did not reach statistical significance [106]. The same pattern of superior cardiac functional recovery was seen when freshly isolated SVFs, instead of their cultured counterparts, were applied to the pig AMI model [105]. Indeed, Alt et al. found that at 8-week follow up to repetitive intracoronary injections of a suspension of uncultured SVFs containing 1.5 × 10^6^ cells/kg a 20% reduction of myocardial perfusion defect was observed, an increase in both myocardial salvage index and ejection fraction, relative to control animals [105]. Given that there is still much debate regarding the question of whether ASCs or SVFs would be more beneficial in therapy, the urgent need to conduct comparable studies seems reasonable.

In the case of ischemic heart injury, as in other types of tissue-specific injury, the ASC delivery route has been tested. Rigol et al. [107] compared the effect of intracoronary and transendocardial administration of autologous ASCs in a porcine model of AMI. At 3 weeks post-treatment, engrafted cells were localized in the border zone of the infarct and close to vessels, regardless of the pathway of their delivery. Similarly, in both experimental groups immunofluorescence analysis demonstrated that some of the implanted cells expressed α-SMA, the marker of smooth muscle cells. However, the total vessel count using histochemical staining for vWF and α-SMA demonstrated that intracoronary infusion was more effective in increasing neovascularization than ASCs injection directly into the affected area [107]. Consistent with this study, Fotuhi et al. [131] adapted a pig AMI model to investigate the effect of intravenous ASCs delivery on heart electrophysiology. As an analogue to clinical practice, at the 8-week follow up, the animals underwent a programmed stimulation protocol for the induction of ventricular arrhythmias. The results indicated that the cell administration method via intravenous infusion did not lead to acute ventricular arrhythmia, bradycardia, or conduction block, so the ASC-based therapy showed a lack of electrophysiological consequences in the porcine AMI model.

Obtaining a sustained effect of cell-based therapy also requires optimization of the timing of cell administration. Substantial cardiac differences were observed between pigs that received ASCs at 15 min and at 7 days after AMI induction [132]. Immunostaining of VEGF, vWF, and α-SMA revealed a significantly higher number of vessels in the infarct border zone in samples collected from animals treated with ASCs 15 min after reperfusion than in pigs who received ASCs 7 days after AMI. Moreover, this study also provided some important data regarding the host immune response, as allogenic ASCs were applied to treat the cardiac artery following reperfusion. At the 3-week follow up, the percentage of area occupied by CD3+ cells was significantly greater in a group that was administered ASCs 15 min after coronary reperfusion, compared with those treated 7 days later. In addition, the formation of donor-specific antibodies in 33.3% of animals treated with ASCs supported the conclusion that allogenic ASCs are not absolutely immune-privileged [132].

A remarkable advantage of the use of large, relatively long-lived animals is the ability to have prolonged time for observation of a therapeutic outcome and possible side effects. In the work performed by Mazo et al. [108], the effect of transendocardial injection of ASCs into a minipig model of MI was analyzed 3 months after transplantation. Several significant long-lasting improvements in cardiac function were observed compared to the medium control, including increases in cardiac contractility and left ventricular ejection fraction (LVEF) and prevention of worsening ventricle geometry, manifested by the significant decrease in end-systolic diameter (ESD) and both end-diastolic (EDV) and end-systolic (ESV) volume. Afterwards, tissue fibrosis (collagen content), vessel density, and myocyte hypertrophy in the border zone of the infarct were measured based on quantification of Sirius Red staining, α-SMA, lectin-I, and laminin, respectively. The results revealed a reduction in fibrosis and cardiomyocyte hypertrophy, accompanied by a significant increase in blood vessel density. Particularly interesting was the observation that ASC transplantation provided an increase in the number of vessels positive for matrix metalloproteinase (MMP) inhibitors (TIMPs), such as TIMP1 and TIMP2, in the infarct border zone. Additionally, a decrease in the activity of MMP2 was found by zymography in serum collected from ASC-treated pigs. These findings clearly show that ASCs might participate in the correction of the imbalance in the MMP/TIMP system that is observed after MI; therefore, they prevent improper remodeling of the heart and suppress scar tissue formation [108]. It is noteworthy that the authors reported a lack of long-term cell engraftment that, analogous to other studies, indicates that the primary mechanism exerted by ASCs on damaged tissue relies on cell secretory functions [133, 134].

### Musculoskeletal System

Much of the interest in the veterinary field has focused on the use of ASCs for musculoskeletal injuries such as tendon, bone, and cartilage lesions (reviewed in [135]) **[**Table 2]. In the vast majority of cases, these defects are associated with failure to return to the previous levels of physical activity along with considerable rates of re-injury, which in horses ranges from 16% to 53% [136, 137]. In practice, the orthopedic injuries provide career-ending conditions in the sport animals.

A major cause of injury in racehorses and in horses used for any discipline is tendinitis of superficial digital flexor tendon (SDFT) [110]. Following injury, the tendon tissue is spontaneously repaired by scar tissue formation; however, the scar is functionally deficient and characterized by lower mechanical strength, reduced elasticity, and increased stiffness [110]. In addition, the healing of tendons is painful, long-lasting, and associated with high costs of treatment. The reported duration of recovery period is between 9 and 14 months and can be even prolonged up to18 months or longer [137–139]. These common complications provide important consequences for the animal in terms of reduced performance [110, 139].

Considering the fact that in several published works cell therapies using MSCs from distinct sources, including bone marrow and umbilical cord blood, have shown promising results in the context of musculoskeletal disease, currently there is growing interest in using autologous ASCs as an alternative therapeutic option in clinical practice [140, 141]. Despite the fact that in veterinary medicine, cell-based therapy is a clinical reality, within the equine orthopedic field, ASCs have been used primarily in experimentally induced tissue defects with a very limited number of reports describing spontaneously occurring clinical cases. An initial study by Del Bue et al. [70] evaluated the clinical effect of delivery of allogeneic ASCs in association with autologous PRP into naturally occurring SDFT lesions in equine athletes. Although the report lacks details of the experimental procedure and no control (untreated) animals were included in the trial, they observed satisfactory recovery at more than 23 weeks follow up and return to activity in 14 of 16 treated horses [69]. Likewise, Ricco et al. [109] showed short- and long-term outcomes (0–1 and 24 months, respectively) of 19 clinical cases of horses affected by acute or sub-acute SDFT that received allogenic ASCs combined with autologous PRP used as a cell carrier. Each lesion underwent a single injection of 2–6 mL cell suspension (2.0 × l0^6^ cells/mL) based on the lesion size. No detrimental effects that could manifest by acute tendon reaction, lameness, local swelling, or heat were found in the short term after cell transplantation. Furthermore, long-term ultrasonographic examination of the tendon revealed lack of abnormal tissue outgrowth within and around the ASC implantation site. This evidence of improved healing was supported by ultrasound images demonstrating greater echogenicity of the tendon structures emerging 60–90 days after treatment. Equally important, at 24 months follow up, 17 of 19 horses (89.5%) regained their previous activity [109]. In another project, Guercio et al. [110] used 1.0 × 10^6^ cells in 5–10 mL of PRP for a treatment of veterinary patients with spontaneous and acute SDFT in the forelimb. Consistent with the aforementioned studies, in this work the authors indicated at 3 months post-treatment some benefits related to tissue organization, based on ultrasound analysis demonstrating that characteristics indicative of a repair process resulted in formation of tissue morphologically comparable, in terms of size and alignment of the fibers, to healthy tissue. Finally, this investigation showed that 7 of 9 horses resumed their normal performance after several months of ASC transplantation [110]. However, it should be said that in the studies discussed here, the stem cell-based therapies were accompanied by rehabilitative physiotherapy, which provides essential support for the proper remodeling of the tendon and leads to restoring tissue functionality (reviewed in [142]). Thus, reported improvements in the health of equine patients might be attributed, at least in part, to the progressive rehabilitation programs. In addition, such clinical studies do not provide sufficient conclusions about the outcome of ASC therapy, primarily due to the small number of animals treated and lack of controls. On the other hand, in contrast to designed in vivo studies/trials that mainly involve selective, homogenous groups of animals, the significant advantage of such clinical cases is that they comprised spontaneous lesions in animals heterogeneous for age, sex, diet, and activity, and hence, the therapeutic results obtained represent a broad range of naturally occurring variables, and in clinical practice this allows for better adjustment of therapeutic procedures to individual clinical situations. Nevertheless, numerous studies have demonstrated the use of animals as a model for diseases with experimentally induced orthopedic lesions [143, 144]. In some of these studies, the therapeutic potential of ASCs is demonstrated by less inflammatory infiltrates to the affected tissue, increased blood flow, or well defined collagen-fiber organization compared to the untreated control group [143, 144]. Therapeutic benefits from application of stem cell types in horses are reviewed in greater detail by Shojaee [22] and De Schauwer [145].

### Endometrosis

ASCs can be considered a promising candidate for cell therapies in equine endometrosis, a chronic degenerative condition of the endometrium **[**Table 2**]** [111, 112]. Endometrosis is defined as active or inactive fibrosis around the endometrial glands and in the stroma that is often associated with pathological changes in the endometrial glands within fibrotic foci [146, 147]. Equine endometrosis causes alterations in endometrial function manifested by changes in its histology, ultimately leading to changes in the uterine microenvironment, ovarian cycle, and early pregnancy dysfunction [148–150]. Endometrial fibrosis is a serious problem in horse reproduction and poses a great economic loss to the horse-breeding industry. Currently, no effective treatment is available for this condition. Mambelli et al. [111] transplanted in utero 2 × 10^7^ fluorescence-labelled mare ASCs (1 × 10^7^ cells in 10 mL 0.9% sodium chloride per each uterine horn) using a simple technique, similar to artificial insemination. The cells were detected in uteri at 7 and 21 days post-delivery and widely distributed in the glandular and periglandular area. At the 60-day follow up, there was no fluorescent signal detected in the uterine specimens. Having adapted this experimental procedure, the same group found in a separate study that allogenic ASCs induced early remodeling of endometrial tissue [112]. This process was manifested by the changes in the expression profile of endometrosis-associated proteins such as laminin, vimentin, Ki-67 antigen, α-SMA, and cytokeratin 18 (CK18). Indeed, upon ASCs delivery no co-expression of vimentin and CK18 was observed, and expression of α-SMA was no longer observed at day 7 post-transplantation. Moreover, the number Ki-67-positive cells was significantly higher in animals treated with ASCs in comparison with untreated mares [112]. These unique studies are promising for the development of therapy to treat equine endometrosis. However, particularly interesting from the perspective of the present review is the fact that the authors used cells that had been cryopreserved in liquid nitrogen for two years and, after thawing, were directly applied to the animals without additional cultivation in vitro [112]. This approach clearly indicates feasibility of cryopreserved ASCs and advocates for their availability as an off-the-shelf product that will allow veterinarians to use them directly at the point of care.

## Conclusions and Future Perspectives

This review demonstrates the current status of knowledge in terms of isolation techniques, culture, and multilevel characteristics of ASCs from farm animals, with particular attention focused on pigs and horses. Moreover, we provide a perspective on the general principles of ASC-based therapy in pig and horse models of human diseases, as well as highlighting the importance of using animal ASCs in veterinary practice. Although employing large animals for research on ASC therapeutic application generates higher costs due to animal size and husbandry needs compared to smaller laboratory animal models, their importance in the field of human diseases is evident as they have greater predictive ability. Thus, the ability to apply human-like settings to animal models increases the chances of bench findings translating to effective treatments.

It is important to recognize and appreciate the fact that the exploration of cellular therapies with the use of large animals can be valuable in establishing alternative medical approaches needed in both preclinical animal models of human diseases and for treating a wide range of conditions that affect animals. As we have discussed, the multitude of different protocols for ASC isolation and the diversity of their delivery routes provide evidence of how much interest in ASC-based therapy in farm animals exists today. However, there are still many issues that need to be addressed when considering ASC-based therapy. First of all, the mechanisms of action through which ASCs enhance healing or attenuate disease are incompletely understood. Although the beneficial properties of ASCs are shown to be related to their immune suppression, anti-inflammatory, and pro-angiogenic roles, knowledge about the specific pathways through which ASCs achieve their pro-repair/pro-regenerative goals needs substantial research effort. Second, further investigations are required to standardize the protocols of the cell acquisition and therapy model in order to ensure safety, efficacy, and consistency for ASC therapy in animal patients. Currently, there are a multitude of different protocols with regard to patient vs. donor source, cell isolation technique, cell culture technique, MSC activation status, MSC dose, and route of administration, making it difficult to compare and choose the most optimal direction of ASC therapeutic development. The selection of the best suited method would guarantee good cell survival, wide distribution, and successful homing of cells in injured tissues, and finally, it must lead to satisfactory therapeutic outcome. Thirdly, in contrast to the slower translation of cellular therapies in human medicine, in the veterinary field such treatments have been rapidly commercialized from experimental models to mainstream clinical practice. Hence, the use of ASCs in veterinary practice requires strict regulatory procedures to ensure efficacy and safety of stem cell-based product applications.

There is no doubt that the use of ASC therapy in veterinary practice has incredible potential in advancing cure options for domestic animals. However, we should be aware of the limitations regarding these therapies, and more in-depth research is necessary to fully understand the potential of ASCs.
